# Fenestration and Dehiscence in Human Maxillary Alveolar Bone: An In Silico Study Using the Finite Element Method

**DOI:** 10.7759/cureus.50772

**Published:** 2023-12-19

**Authors:** Camila C Furlan, Alexandre R Freire, Beatriz C Ferreira-Pileggi, Felippe B Prado, Ana Cláudia Rossi

**Affiliations:** 1 Biosciences, Piracicaba Dental School, University of Campinas (UNICAMP), Piracicaba, BRA

**Keywords:** finite element method, biomechanics, alveolar bone, dehiscence, fenestration

## Abstract

Introduction: Fenestration and dehiscence are alveolar bone defects. Although not considered a pathology, these alveolar bone defects end up influencing dental treatment, such as surgeries, mainly periodontal, and therefore must be considered during treatment planning. However, currently, little is known about the biomechanical origin of these bone formations. The aim of the study was to use the finite element method (FEM) to test hypotheses of predictive factors for fenestrations and dehiscence in human alveolar bone.

Methods: A FEM simulation of the action of functional, parafunctional, and orthodontic occlusal loads on the upper central incisor and upper canine was performed. For the simulation, a three-dimensional model of an adult human skull, fully dented and with intact bone structure, was constructed from computed tomography images. The buccal alveolar bone lamina was evaluated considering the calculation of equivalent stresses, as well as maximum principal stresses.

Results: The action of functional and parafunctional forces on the incisal edges and the orthodontic force on the buccal face of the upper central incisor and upper canine teeth generated tensions at different levels of magnitude in the buccal bone lamina, varying in regions, at all levels of strength. Changing levels of force magnitude resulted in variations in relation to the level of deformation.

Conclusion: The computational simulation using the FEM was able to identify a difference in stress in the alveolar bone tissue in each of the applied forces. The difference in stresses obtained may suggest the formation of dehiscence or fenestration in the region studied.

## Introduction

Fenestration and dehiscence are alveolar bone defects. Fenestration consists of an absence of part of the lingual or buccal alveolar bone lamina so that the root becomes exposed; however, the marginal bone tissue continues to exist. Dehiscence, in turn, can be defined as an increase in the distance between the cementoenamel junction and the alveolar bone crest [1].

The alveolar process is one of the anatomical components of the periodontium characterized as an extension of the body of the maxilla and mandible that relates to the teeth, forming the alveoli. The morphology of the socket is dependent on the teeth and can acquire two types of interruption of the bone lamina, known as dehiscence or fenestrations; however, the etiology of these conditions, as well as the clinical cult, are not well understood [2].

The etiology of dehiscence and fenestration may be involved with the curvature of the dental root, trauma, positioning of the teeth, force of occlusion, and occlusal movement, in addition to the thickness of the alveolar bone [2-4]. Based on the results of studies on the incidence and prevalence of the presence of dehiscence and fenestrations, the upper and lower incisors, upper canines, upper premolars, and upper molars are teeth commonly associated with these conditions [5].

Although not considered a pathology, these alveolar bone defects end up influencing dental treatment, such as surgeries, mainly periodontal, and therefore must be considered during treatment planning [1].

These bone lesions are commonly present in malocclusions, mainly in the anterior region of class III malocclusion, affecting orthodontic treatment, which must use the morphology of the alveolar bone lamina to define the procedures to be performed, since dehiscence and fenestration, during orthodontic treatment, can lead to gingival recession and alveolar bone loss [6]. For safe tooth movement in cases of dehiscence and fenestration, due to the thin thickness of the alveolar bone plates, it is important to consider grafting, increasing the bone surface over the root [7,8]. Sun et al. [8] evaluated the changes in alveolar dehiscence and fenestration after augmented corticotomy-assisted orthodontic treatment on cone-beam computed tomography compared with traditional pre-surgical orthodontics. They concluded that for skeletal class III patients, augmented corticotomy-assisted orthodontic treatment is a promising method to improve the alveolar bone dehiscence and fenestration of potential to protect both lower and upper anterior teeth from dehiscence.

The exposure of the alveolar bone usually occurs during periodontal and oral surgery, and the presence of fenestrations and dehiscence is of great importance, as they may complicate the outcome during the healing process. Special conditions must be considered regarding the current trend in oral implant dentistry - the flapless approach, once, through this technique, it is not always possible to perceive the presence of fenestrations and dehiscence, a situation that can negatively affect both osseointegration and the aesthetic result [9,10]. More research needs to be done related to the influence of dehiscence and fenestrations to mechanically understand the rate and pattern of bone loss and alveolar bone healing.

The finite element method (FEM) is a numerical evaluation based on mathematical equations to determine data on stresses, deformations, and displacements in computationally elaborated models, with great importance in studies for understanding craniofacial skeleton biomechanics and is, therefore, considered an effective method for biomechanical evaluation of structures with complex geometries, such as bony structures [11-13].

The aim of this study was to create three-dimensional models of alveolar bone and teeth simulating dehiscence and fenestration conditions of human alveolar bone and use the FEM to test the hypothesis that orthodontic forces and occlusal overloads can act as predictive factors for the appearance of dehiscence and fenestrations in the alveolar bone of the maxillary central incisor and maxillary canine teeth, given the importance of knowing these bone formations in dental treatment.

## Materials and methods

Sample

A computational model of an adult human skull was used as a sample in this study. The model was built from images of computed tomography of a dry skull, with all bone structures intact and fully dented. The selected tomography images belong to one (one) male skull, aged between 30 and 40 years.

The computed tomography of the human skull selected for the present research came from the osteological and tomographic biobank “Prof. Dr. Eduardo Daruge” of the Piracicaba Dental School at the University of Campinas (UNICAMP).

Inclusion and exclusion criteria of the sample

The CT scan of the skull selected to integrate the sample has intact bone structures, without bone or surgical pathologies, and has all upper and lower teeth. The skull CT scans with any anatomical abnormalities in the region of interest, as well as individuals with implants, plates, screws, or any other metal artifact near the region were not selected to be the sample skull.

Segmentation of tomographic images for the construction of three-dimensional models

**Figure 1:**
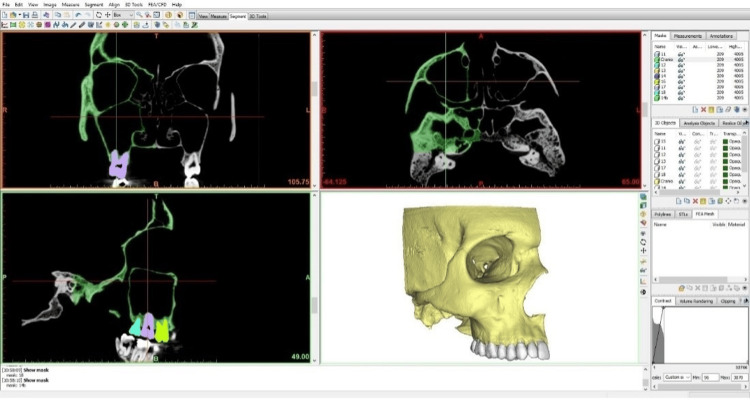
Tomographic images being segmented in the Materialise MIMICS Academic Research v18 software (Materialise, Leuven, Belgium).

Initially, the images from computed tomography of the skull were imported into the Materialise MIMICS Academic Research v18 software (Materialise, Leuven, Belgium) to perform the segmentation of the bony structure and teeth (Figure 1). To perform the segmentation, the pixel marking tool was used, based on the threshold of grayscale values. This threshold was configured in ranges of values corresponding to the specific marking for bony structure and teeth.

After segmentation, the pixels marked for each structure were converted into three-dimensional virtual stereolithographic (STL) surfaces, which were transferred to the software Materialise 3-Matic Academic Research v10 (Materialise, Leuven, Belgium). In this step, the STL surfaces were optimized to correct geometric errors, preserving the anatomy of the structures. After correcting the STL surfaces, they were converted into volumetric meshes, formed by tetrahedral elements, thus forming the finite element mesh of each structure. An evaluation of the geometric quality of the meshes was carried out (where it is classified in values from 0 to 1, with 1 corresponding to the best quality). In addition, the preservation of the anatomy was evaluated based on the qualitative analysis of the anatomical accidents.

**Figure 2:**
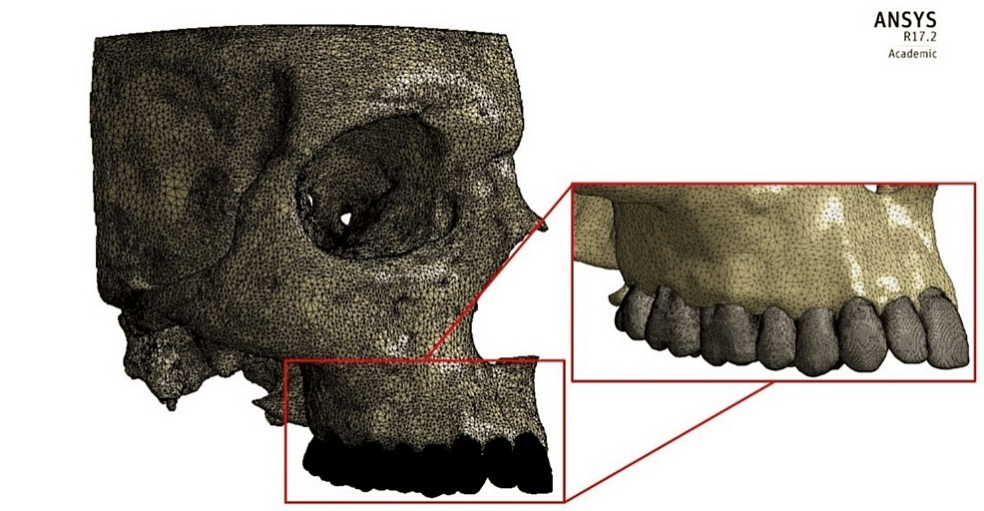
Finite element mesh. Software Ansys Academic v17.2 (Ansys Inc., Cannonsburg, PA).

Thus, the finite element mesh of the central incisor and upper canine teeth and their bony structures was constructed (Figure 2).

Computer simulation using the finite element method

After building the geometry, simulations were performed using Ansys Academic v17.2 software (Ansys Inc., Cannonsburg, PA). The simulations were configured for the action of an orthodontic force of 1.28 N on the center of the buccal surfaces of the upper central incisor and upper canine, in addition to functional forces of 100N and parafunctional forces of 500N on the incisal edges of the same teeth.

The finite element models were imported, and each structure received the characterization of the mechanical properties (Table 1), which were obtained through previous studies present in the literature. Stiffness properties were applied, and the structures were considered formed by linear and elastic materials.

**Table 1:** Mechanical properties of structures. References [14,15].

Anatomical structure	Modulus of elasticity	Poisson coefficient
Bone	14000	0.3
Tooth	19600	0.3

To characterize the stability and positioning of the skull during occlusal action, boundary conditions were applied with displacement constraints in all axes at the edges of the finite element model.

Data analysis

Von-Mises equivalent stresses (VM) and maximum principal stresses (MP) were calculated, both calculated in megapascals (MPa), for analysis of mechanical stimuli distributed over the regions of interest (buccal alveolar bone) after application of functional forces, parafunctional and orthodontic. The alveolar bone was observed according to the regions referring to the root levels of the corresponding tooth (cervical, middle, and apical levels), both on the external and internal surfaces.

## Results

**Figure 3:**
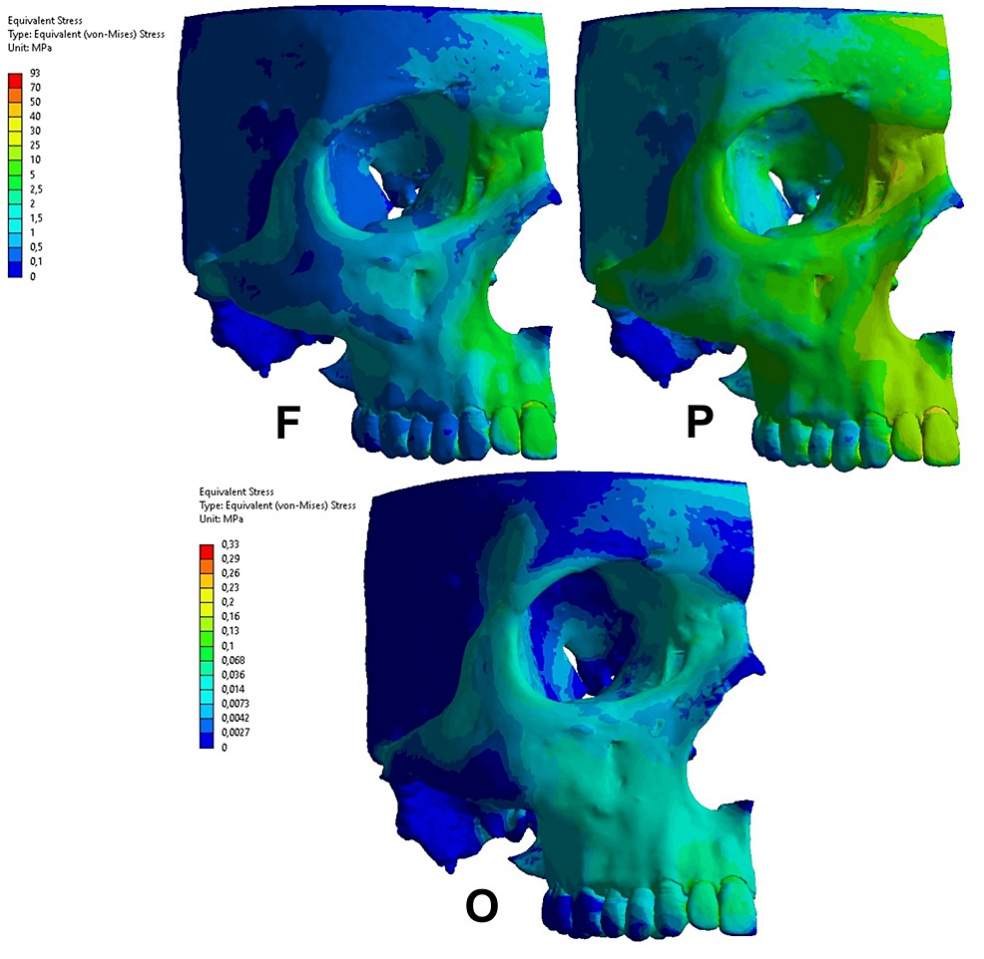
Equivalent von Mises stress in the functional condition (F), parafunctional condition (P), and orthodontic condition (O) on the upper central incisor. The same color scale was used from the F and P conditions. Regarding the orthodontic condition, a different color scale was used due to the difference in the magnitude.

**Figure 4:**
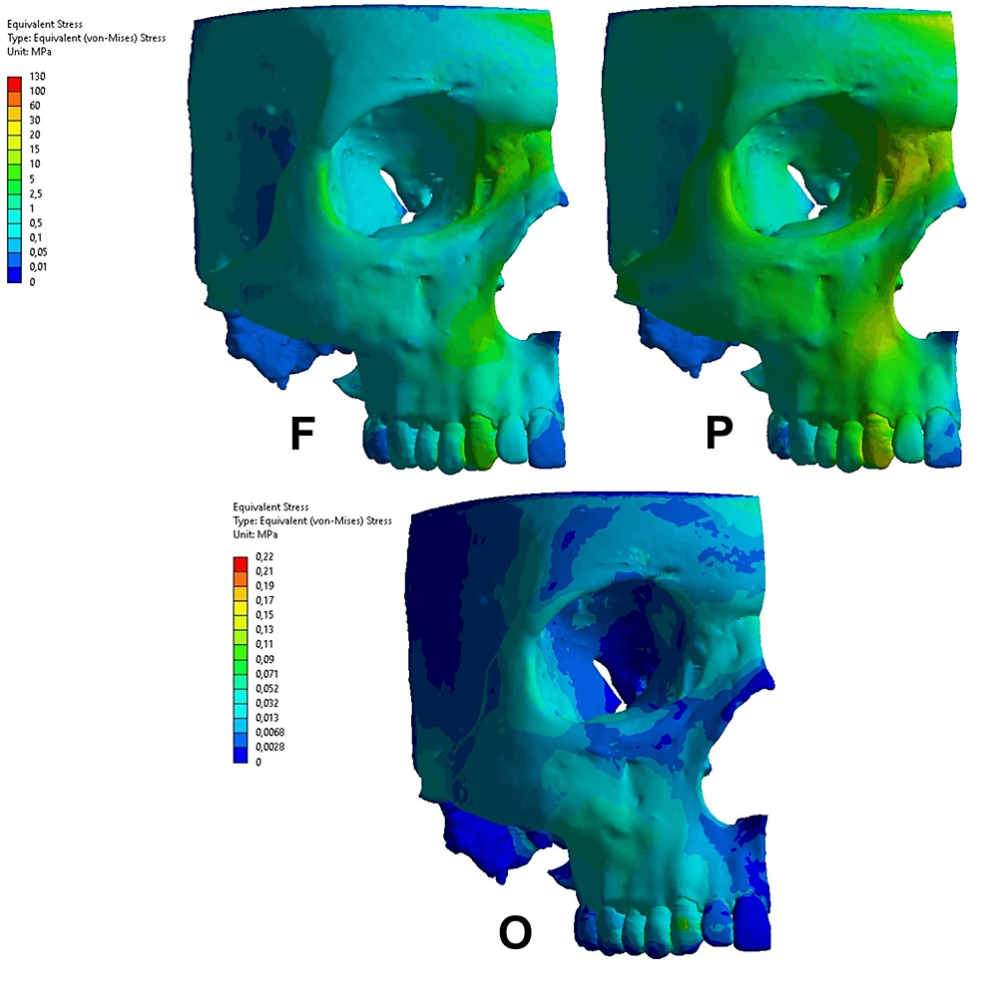
Equivalent von Mises stress in the functional condition (F), parafunctional condition (P), and orthodontic condition (O) on the upper canine tooth. The same color scale was used from the F and P conditions. Regarding the orthodontic condition, a different color scale was used due to the difference in the magnitude.

**Figure 5:**
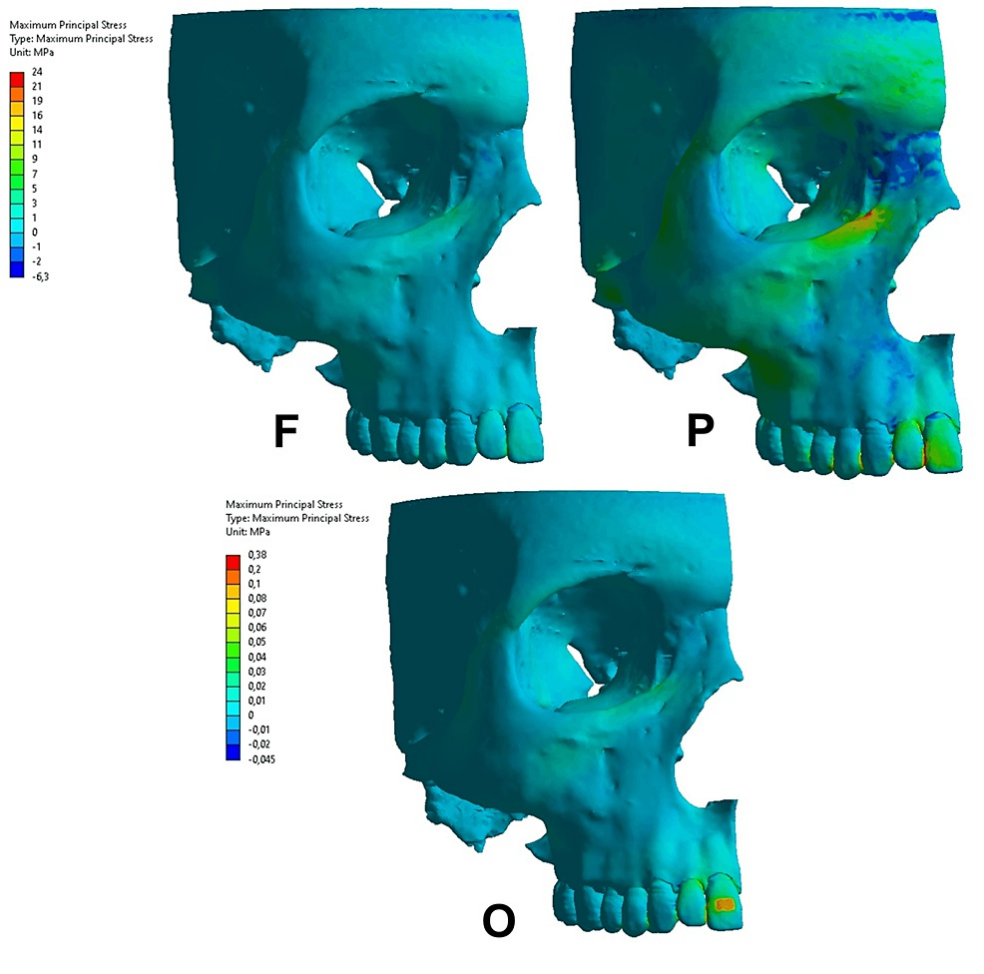
Maximum principal stress in the functional condition (F), parafunctional condition (P), and orthodontic condition (O) on the upper central incisor. The same color scale was used from the F and P conditions. Regarding the orthodontic condition, a different color scale was used due to the difference in the magnitude.

**Figure 6:**
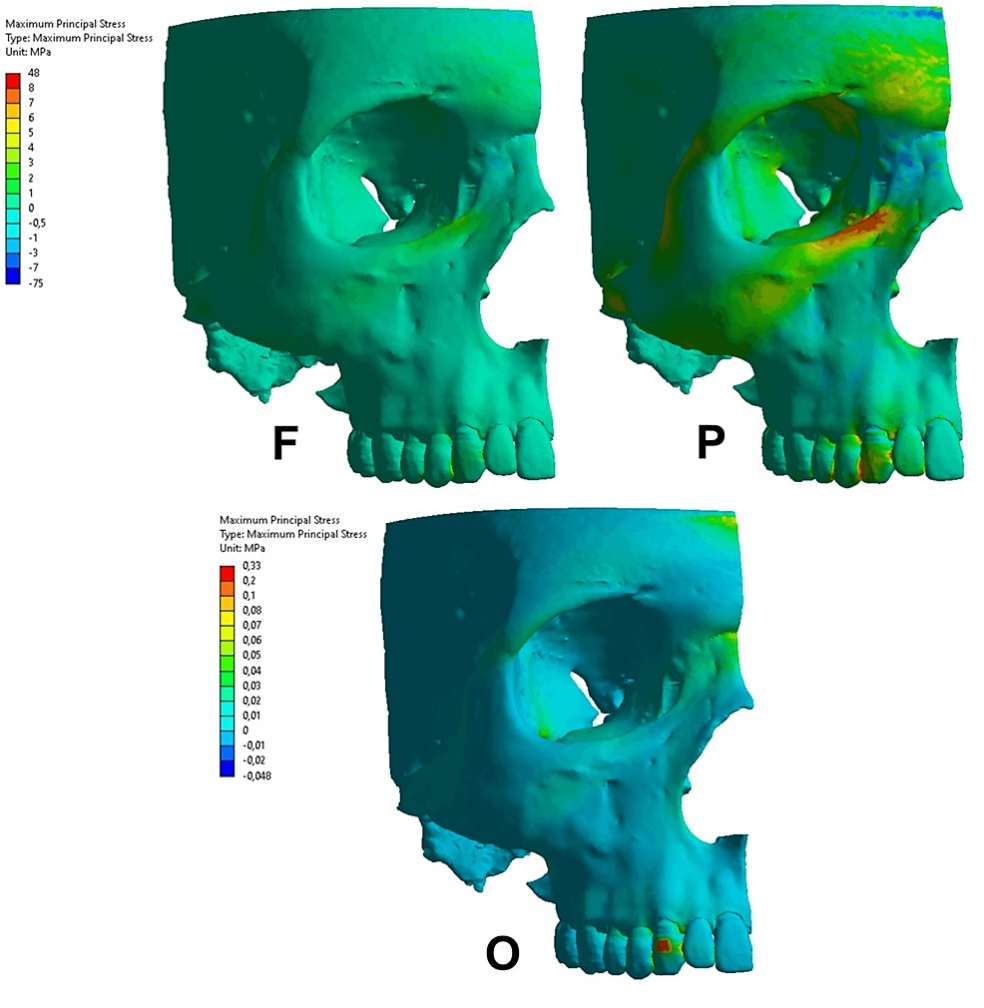
Maximum principal stress in the functional condition (F), parafunctional condition (P), and orthodontic condition (O) on the upper canine tooth. The same color scale was used from the F and P conditions. Regarding the orthodontic condition, a different color scale was used due to the difference in the magnitude.

The figures show (Figures 3-6) the more concentrated stress areas according to a color scale, which presents the interval of stress values. All the results were observed and described in comparison to the functional condition. In addition, the stress areas in the alveolar bone were observed following the dental root level as a reference, i.e., cervical, middle, and apical levels.

Equivalent von Mises stress

The VM stress distribution presented different patterns regarding the action of functional, parafunctional, and orthodontic forces. Regarding the parafunctional and orthodontic forces on the upper central incisor (Figure 3), the VM distribution presented different stress concentrations. A VM stress was observed in the alveolar bone ranging from 2 to 5 MPa in the functional condition, where the higher concentration occurred at the cervical level. The parafunctional condition resulted in a VM stress interval ranging from 10 to 25 MPa, presenting a 5x higher stress concentration in the alveolar bone at all levels. Regarding the orthodontic condition, a lower stress magnitude was observed ranging from 0.014 to 0.068 MPa in the cervical level of the alveolar bone.

In the upper canine (Figure 4), the parafunctional condition revealed more stress concentration at the apical third level. In the upper canine, the results also presented differences in the parafunctional and orthodontic conditions, compared to functional conditions. The functional condition resulted in VM stress ranging from 1 to 5 MPa, where the higher stress concentration occurred in the apical root level in the alveolar bone. The parafunctional condition presented a similar stress distribution regarding the area of stress concentration, but the stress magnitude was higher ranging from 5 to 30 MPa. The orthodontic condition resulted in stress magnitude ranging from 0.013 to 0.032 MPa and the higher stress concentration occurred in the cervical and middle levels.

Maximum principal stresses

The MP was observed according to the areas that presented positive values (tensile stress) or negative values (compressive stress). The functional condition on the upper central incisor (Figure 5) resulted in tensile stress, ranging from 0 to 1 MPa throughout the alveolar bone. The parafunctional condition presented a compressive area on the alveolar margin, ranging from -1 to 0 MPa.

The MP on the upper canine region (Figure 6) presented different results in the functional, parafunctional, and orthodontic conditions. In functional conditions, the MP presented tensile stress at all levels of the alveolar bone with values between 0 and 1 MPa. The parafunctional condition resulted in greater tensile magnitude at the level of the cervical third with values between 1 and 3 MPa, lower magnitude in the middle third ranging from 0 to 1 MPa, and presence of compressive stress at the apical level, ranging from -0.5 to 0 MPa. The orthodontic condition also presented variations regarding the tensile and compressive stress. At the cervical level, compressive stress occurred in the distal region of the alveolar bone, ranging from -0.01 to 0 MPa, and tensile stress in the mesial region, ranging from 0.01 to 0.02 MPa. In the middle level, tensile stress was observed ranging from 0.01 to 0.02 MPa. In the apical level, compressive and tensile stress occurred, with values ranging from -0.01 to 0 MPa and 0 to 0.01 MPa, respectively.

## Discussion

To understand dehiscence and fenestration, it is important to consider the mechanical events that can influence alveolar bone remodeling. The mechanostat theory discussed by Henneman et al. [16] describes the change in blood flow, which can occur during mechanical variations in conditions other than functional, such as orthodontic force; there is cell death, reabsorption of hyalinized tissue by macrophages, and bone tissue by osteoclasts since this change in vascularization ends up resulting in the release of cytokines, neurotransmitters, growth factors, colony-stimulating factors, and other molecules that end up recruiting osteoclasts and other inflammatory cells to reabsorb local bone tissue [17].

According to Omi and Mishina [18], the mechanostat theory is applied to the long bones and it is uncertain if it is applicable to the alveolar bone in response to dynamic loading as seen in masticatory conditions and/or parafunctional and orthodontics forces. It must also be considered that turnover in the alveolar bone is faster than that in the other skeletal bones [19]. Variations in mechanical stimuli, such as stress on the alveolar bone structure, have been related to changes in the bone remodeling process, in experimental observations associated with computational simulations to simulate tooth extraction [20] and premature contact [21]. Therefore, the results observed in FEM become increasingly compatible with interpretations related to the biological response.

Grimoud et al. [22] presented a list of predictive factors for alveolar bone resorption. By analyzing the distribution and incidence of alveolar bone resorption over time in a medieval French population, along with nine other studies, they found that the position and function of the teeth were the most important. The anterior teeth were most affected, bone resorption was more common on the buccal versus lingual surfaces, fenestration was also more common in the maxilla, and dehiscence was in the mandible. These authors concluded that these patterns do not vary over time or space and therefore provide predictive factors for dental health professionals to improve patient recovery and post-oral treatment success.

In the present study, the forces applied on upper incisors, both parafunctional and orthodontic conditions, it was suggested that the stress concentration is related to the appearance of dehiscence. A possible explanation could be based on the shape of the root, since the root of the central incisor is conical and short, the surrounding bone is trabecular and uniform, which causes a more homogeneous dissipation of tension. In the upper canine, the parafunctional condition revealed more stress concentration at the apical third level. In this case, this condition suggests the appearance of fenestration. In this situation, it is possible to observe that the stresses followed the dissipation through the canine pillar [12,23]. The results of Freire et al.'s [23] study may explain the concentration of stress at the apical third level of the upper canine root. The authors used a FEM of an adult male human facial skeleton to evaluate the distribution of von Mises and principal stresses in the canine pillar. They simulate the action of the jaw elevator muscles. They found high von Mises stresses in the inferolateral corner of the piriform aperture where principal stresses are compressive collaborating with our findings.

The study by Nalbantoğlu and Yanık [24] evaluated the buccal bone thickness (BT) and compared the prevalence of bone fenestration and dehiscence in anterior maxillary teeth using cone-beam computed tomography. They evaluated 300 images of maxillary anterior teeth. The BT was measured at the bone crest, 3, 6, and 9 mm from the bone crest, and apical. The authors found the prevalence of fenestration and dehiscence in the anterior maxilla were 35.66% and 20%, respectively. Corroborating our study, they concluded that the BT of the upper canine was thinnest compared with incisors at the level of apex, which is consistent with our results that the parafunctional condition revealed more stress concentration at the apical third level. In this case, this condition suggests the appearance of fenestration.

The limitations of the present study are that it is a generic computational model, and we did not consider systemic variations that affect bone quality (for example, osteoporosis), or the condition of oral health. General factors of variation, such as sex, age, and ethnicity, were also not considered. The present study is important to guide clinicians in planning orthodontic treatments that require extensive anatomical and biomechanical knowledge of the alveolar bone to predict the effects of orthodontic movement.

## Conclusions

In conclusion, the relevant point of the present study is that the computational simulation using the FEM was able to identify a difference in stress in the alveolar bone tissue in each of the applied forces. The time and frequency of forces are important issues for a better understanding of the effect of these predictive factors in the analyses so that bone remodeling can be confirmed and whether there would be actual formation of dehiscence and fenestrations. The study of the type of mechanical force that causes fenestrations and dehiscence helps to clinically understand the health of the periodontium and clinically assist in the planning of treatments such as graft placement for oral implant surgery.

The present study explores the concept of biomechanics in a generic model, which will serve as a basis for future studies and clinical applications. This study generically addresses the biomechanical context whose basis will serve for future studies involving different population variations such as sex, age, and ethnicity.
